# 6‐year change in high sensitivity cardiac troponin T and the risk of atrial fibrillation in the Atherosclerosis Risk in Communities cohort

**DOI:** 10.1002/clc.23727

**Published:** 2021-09-21

**Authors:** Linzi Li, Elizabeth Selvin, Ron C. Hoogeveen, Elsayed Z. Soliman, Lin Y. Chen, Faye L. Norby, Alvaro Alonso

**Affiliations:** ^1^ Department of Epidemiology, Rollins School of Public Health Emory University Atlanta Georgia USA; ^2^ Department of Epidemiology and the Welch Center for Prevention, Epidemiology and Clinical Research Johns Hopkins Bloomberg School of Public Health Baltimore Maryland USA; ^3^ Division of General Internal Medicine, Department of Medicine Johns Hopkins University Baltimore Maryland USA; ^4^ Department of Medicine, Division of Cardiovascular Research Baylor College of Medicine Houston Texas USA; ^5^ Epidemiological Cardiology Research Center, Department of Epidemiology and Prevention Wake Forest School of Medicine Winston‐Salem North Carolina USA; ^6^ Cardiovascular Division, Department of Medicine University of Minnesota Medical School Minneapolis Minnesota USA; ^7^ Department of Cardiology, Smidt Heart Institute Cedars‐Sinai Health System Los Angeles California USA

**Keywords:** atrial fibrillation, hs‐cTnT, risk prediction

## Abstract

**Background:**

Circulating high sensitivity cardiac troponin T (hs‐cTnT) is associated with incidence of atrial fibrillation (AF), but the association of changes in hs‐cTnT over time on incident AF has not been explored.

**Hypothesis:**

Six‐year increase in circulating hs‐cTnT will be associated with increased risk of AF and will contribute to improved prediction of incident AF.

**Methods:**

We conducted a prospective cohort analysis of 8431 participants from the Atherosclerosis Risk in Communities (ARIC) study. hs‐cTnT change was categorized at visit 2 and 4 as undetectable (<5 ng/L), detectable (≥5 ng/L, <14 ng/L), or elevated (≥14 ng/L). We used Cox regression to examine the association between the combination of hs‐cTnT categories at two visits and incident AF. We also assessed the impact of adding absolute hs‐cTnT change on risk discrimination for AF by C‐statistics and net reclassification improvement (NRI).

**Results:**

Over a mean follow‐up of 16.5 years, 1629 incident AF cases were diagnosed. Among participants with undetectable hs‐cTnT at visit 2, the multivariable HR of AF was 1.28 (95% CI 1.12–1.48) among those with detectable or elevated hs‐cTnT at visit 4 compared to those in which hs‐cTnT remained undetectable. Among those with detectable hs‐cTnT at visit 2, compared to those who remained in the detectable hs‐cTnT group, reduction to undetectable at visit 4 was associated with lower risk of AF (HR 0.74, 95% CI 0.59–0.94), while increment to elevated was associated with higher AF risk (HR 1.30, 95% CI 1.01–1.68). Adding hs‐cTnT change to our main model with baseline hs‐cTnT did not result in significant improvement in the C‐statistic or substantial NRI.

**Conclusion:**

Six‐year increase in circulating hs‐cTnT was associated with elevated risk of incident AF.

## 1 INTRODUCTION

Atrial fibrillation (AF) is the most common chronic cardiac arrhythmia in adults, with an estimated prevalence of 2.7 million adults in the United States. In 2030, the prevalence is estimated to rise to 12.1 million.^1^ AF is associated with increased mortality and morbidity, and can lead to severe complications, such as stroke, heart failure (HF), and chronic kidney disease (CKD).^2^, ^3^, ^4^, ^5^ Generally, AF is paroxysmal and occurs asymptomatically, which creates obstacles for the detection of AF. Due to this limitation, investigators have developed and validated risk prediction models for AF, which can assist in identifying high‐risk individuals.^6^, ^7^, ^8^ Addition of novel or repeated and high‐sensitivity risk factor measurements have the potential to enhance the predictive ability of these models.

High sensitivity cardiac troponin T (hs‐cTnT) is a well‐established biomarker of myocardial injury, which is used to diagnose acute myocardial infarction (MI).^9^ A growing body of evidence suggests that MI is associated with AF as preceding or complicating the clinical course of AF.^10^, ^11^, ^12^, ^13^ Prior studies have reported associations between hs‐cTnT and the risk of developing AF, supporting the role of circulating hs‐cTnT as a biomarker of AF risk. In a Japanese general population without apparent cardiovascular disease, circulating hs‐cTnT levels were greater among subjects with AF compared to those without AF.^14^ In the Atherosclerosis Risk in Communities (ARIC) study, the risk of incident AF was 1.16 times higher with 1‐standard deviation increase of ln(hs‐cTnT) level.^15^ In the Cardiovascular Health Study, a large prospective cohort of ambulatory older adults, hs‐cTnT was significantly associated with incident AF beyond traditional risk factors.^16^ Elevated hs‐cTnT levels are also associated with incidence of other cardiovascular and chronic diseases, such as incident HF and CKD,^17^, ^18^ which have similar risk factors and often coexist with AF.^19^, ^20^

The change in biomarker measures over time may be more informative than a 1‐time measurement. We previously found in the ARIC study that change over time in concentrations of N‐terminal pro hormone B‐type natriuretic peptide (NT‐proBNP) was associated with AF risk and improved the ability to predict AF.^21^ Also in the ARIC cohort, 6‐year change in hs‐cTnT was independently associated with all‐cause mortality, HF, and incident coronary heart disease (CHD).^22^ Nevertheless, to our knowledge, little is known about the impact of hs‐cTnT change on the risk of incident AF, as well as the role of hs‐cTnT change in upstream mechanism of developing incident AF. Therefore, we sought to evaluate the association between the change of hs‐cTnT and AF risk.

The ARIC study is a sizable predominantly bi‐racial cohort study in the general US population, with eight follow‐up visits to date. Its repeated measurements of hs‐cTnT in several follow‐ups, rigorously assessed AF endpoints, participant diversity, and long follow‐up provide a unique resource for exploring the role of change in circulating hs‐cTnT as a predictor of incident AF. Taking advantage of the longitudinal data in the ARIC study, we investigated the association of 6‐year change in circulating hs‐cTnT with the risk of incident AF, and determined the value of 6‐year change in hs‐cTnT in the prediction of AF.

## 2 METHODS

### 2.1 Study population

**FIGURE 1:**
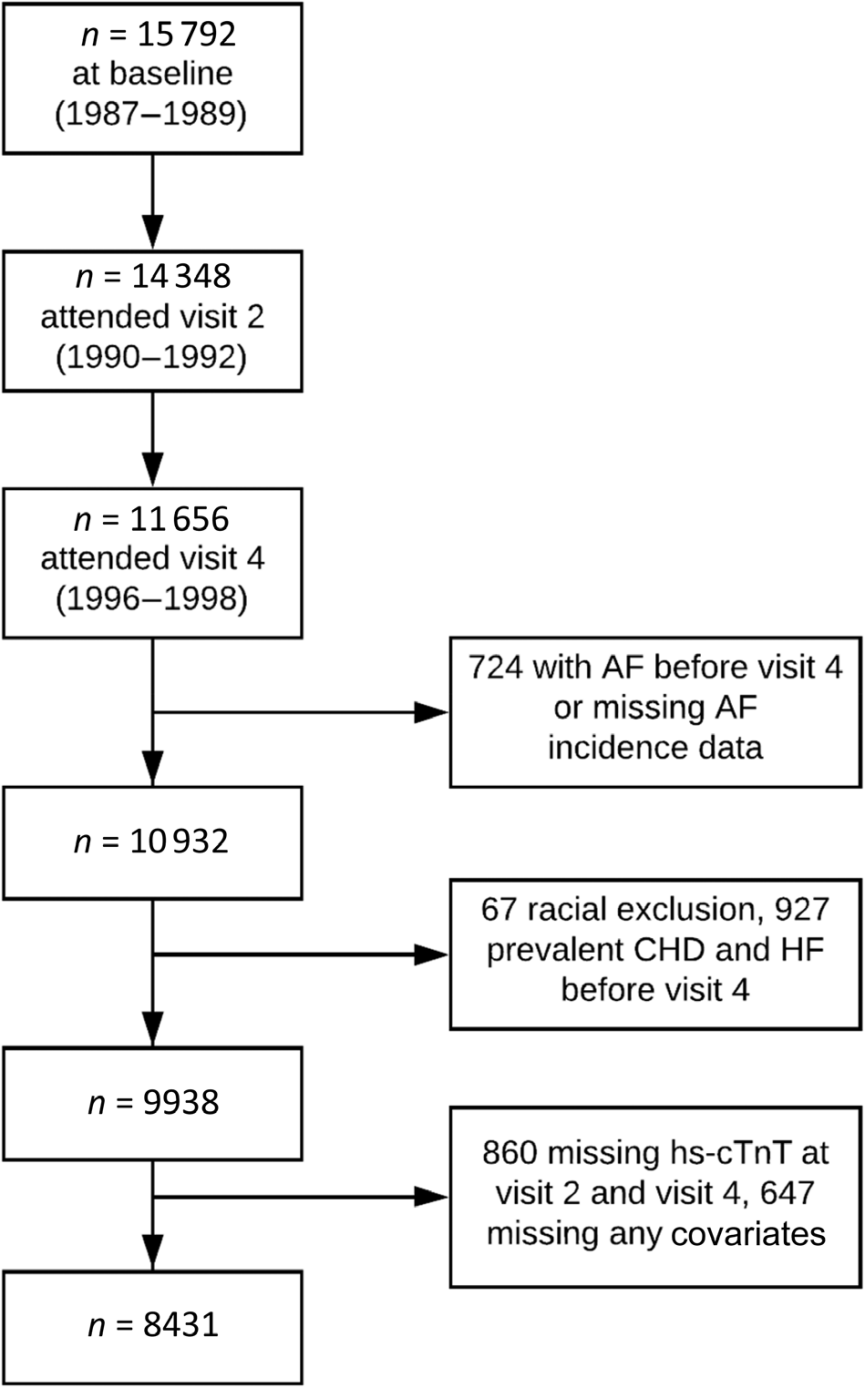
Flowchart of participants inclusion and exclusion

The Atherosclerosis Risk in Communities (ARIC) study is a prospective cohort study conducted in four U.S. communities: Forsyth County, North Carolina; Washington County, Maryland; selected Minneapolis suburbs, Minnesota; and Jackson, Mississippi. The study design has been published elsewhere.^23^ Beginning in 1987, each ARIC field center recruited approximately 4000 study participants aged 45–64 from their community. Between 1987 and 1989, 15 792 individuals were recruited and completed an extensive baseline examination. Of these participants, we included those who had both visit 2 (1990–1992) and visit 4 (1996–1998) hs‐cTnT measurements. We excluded those who had AF, CHD, or HF before visit 4, those with missing values of any covariates, as well as non‐whites from the Minneapolis and Washington County field centers, and individuals other than white or African American in the Forsyth County field center. Finally, 8431 remained as analytic sample in this study (baseline, visit 4 in 1996–1998). Figure 1 shows the flowchart of participants inclusion and exclusion. The study protocol was approved by institutional review boards at participating institutions. All study participants provided written informed consent.

### 2.2 Assessment of high sensitivity cardiac troponin T

Hs‐cTnT was measured in blood samples from visit 2 and visit 4. Concentrations of hs‐cTnT from visit 2 were measured using stored serum samples at the University of Minnesota, using a sandwich immunoassay method with a Roche Elecsys 2010 Analyzer (Roche Diagnostics).^22^ At a mean hs‐cTnT level of 26 and 1990 ng/L, the intra‐assay coefficients of variation (CVs) were 2.1% and 1.0%. At a mean hs‐cTnT level of 25 and 1940 ng/L, the inter‐assay CVs were 6.0% and 3.7%.^22^

Concentrations of hs‐cTnT from visit 4 were measured using stored supernatant plasma samples at Baylor College of Medicine, using an electrochemiluminescence immunoassay with a Roche Cobas e411 analyzer. At a mean hs‐cTnT level of 29 and 2378 ng/L, the intra‐assay CVs were 2.1% and 0.76%. At a mean hs‐cTnT level of 29 and 2378 ng/L, the inter‐assay CVs were 6.9% and 2.6%.^22^ There was no significant differences observed in a formal calibration study which evaluated heterogeneity in hs‐cTnT across specimen type and laboratory.^24^

The measurement assay range is 3–100 000 ng/L. The limit of the blank (LOB) is 3 ng/L, the limit of detection (LOD) is 5 ng/L, and the limit of quantitation (LOQ) is 6 ng/L.^9^, ^25^

### 2.3 Definition of incident atrial fibrillation

We determined AF cases in the ARIC study by three methods. First, during study exams, 12‐lead electrocardiograms (ECGs) data were obtained and transmitted electronically to the ARIC ECG reading center at EPICARE (Wake Forest School of Medicine, Winston‐Salem, NC). The presence of AF or atrial flutter was identified in the ECG by a computer algorithm. To reduce the possibility of missing or misreading episodes of AF, a cardiologist would confirm the computer diagnosis and overread any rhythm disorder other than AF in ECG. Second, participants' hospitalization records including discharge codes were collected by follow‐up phone calls and surveillance of local hospitals. If the ICD‐9‐CM codes 427.31 (AF) or 427.32 (atrial flutter) or ICD‐10‐CM codes I48.x were identified in any given records, then AF was deemed as present. Any AF cases related to open cardiac surgery were excluded. Third, if death certificates listed ICD‐9 427.3 or ICD‐10 I48, then AF was regarded as present. These methods were described in detail in a published paper.^26^ Any participants who were diagnosed with AF through any of these methods was considered to have AF.

We conducted a study to examine the validity of AF ascertainment in ARIC. First, we reviewed hospital discharge records from 125 potential cases of AF based on ICD diagnosis codes, finding that the positive predictive value of these codes was approximately 90% (111 of 125 confirmed). Second, we evaluated sensitivity and specificity of the ICD codes among ARIC participants undergoing surveillance for stroke—the corresponding values were 84% and 98%.^26^

### 2.4 Ascertainment of other covariates

The following variables measured at both visit 2 and visit 4 were used as covariates in this study: age, sex, race, body mass index (BMI), smoking status (current, former, never), drinking status (current, former, never), systolic blood pressure (SBP), low‐density lipoprotein cholesterol (LDLc), high‐density lipoprotein cholesterol (HDLc), ECG p wave terminal force in V1, triglycerides, diabetes history, ECG‐based left ventricular hypertrophy (LVH), use of anti‐hypertension medication, use of lipid lowering medications, c‐reactive protein, NT‐proBNP, estimated glomerular filtration rate (eGFR, measured from both creatinine and cystatin‐C) and study center. SBP was the mean of the last two of three measurements at visit 2 and the mean of two measurements at visit 4. Diabetes was diagnosed as fasting blood glucose ≥126 mg/dl, non‐fasting blood glucose ≥200 mg/dl, use of antidiabetic medication, or a self‐reported physician diagnosis of diabetes. Participants were asked to bring any medications and supplements taken the 2 weeks prior to the exam. Medication use was determined by staff review at the time of the visit. Age, sex, race, smoking status, and drinking status were self‐reported.

### 2.5 Statistical analysis

The main independent variable was the change of hs‐cTnT concentration between visit 2 and visit 4. We first grouped individuals based on combinations of categories of hs‐cTnT at visit 2 and visit 4: undetectable (<5 ng/L), detectable (≥5 ng/L, <14 ng/L), and elevated (≥14 ng/L).^22^ The baseline (visit 4) characteristics of our study population was described according to these categories at visit 2 and visit 4 (mean [SD] for continuous variables and frequency [percentage] for categorical variables). The time of follow‐up was days from visit 4 to incident AF, death, loss to follow‐up or December 31, 2017, whichever happened earlier. The incidence rate of AF was calculated in each group. To examine the association between the change of hs‐cTnT and the risk of incident AF, we used Cox proportional hazards regression to estimate the hazard ratio (HRs) and 95% confidence intervals (CIs), in the following two models: (1) only adjusted for age, sex and race; (2) fully adjusted for all the covariates listed above + log‐transformed visit 2 hs‐cTnT level. We also report the association of incident AF per 1‐unit change of log‐transformed change of hs‐cTnT between visit 2 and visit 4, and as percentage of relative change from the measurement at visit 2. Finally, we explored the predictive ability of change in hs‐cTnT by adding it to the CHARGE‐AF score, which is a validated risk model for predicting incident AF, including information on age, race, height, weight, SBP, DBP, current smoking, antihypertensive medication use, diabetes, HF, MI, LVH by electrocardiogram.^6^ We calculated the change in c‐statistic and the net reclassification index (NRI) to quantify the improvement offered by the change in hs‐cTnT, using the 10‐year risk categories <5%, 5%–10%, and >10% as cutoffs, based on the CHARGE‐AF model.^6^, ^27^, ^28^ Because there have been no established risk 10‐year risk categories for AF, we doubled the 5‐year risk categories in the CHARGE‐AF model <2.5%, 2.5%–5%, >5%.^6^ We also calculated the continuous NRI since the category‐based NRIs are highly dependent on the cutoffs selection and numbers of categories. The continuous NRI is defined by any upward and downward movements in predicted risks and can be used universally.^27^, ^28^

In a sensitivity analysis, to minimize the effect of missing data of participants who were dead or loss to follow up, we used multiple imputation by chained equations (MICE) to impute all missing covariates and hs‐cTnT at visit 2 and visit 4 in a multivariable model including completely collected covariates. We re‐run the models after creating 20 additional datasets. All the analyses were completed with SAS statistical software (v. 9.4, SAS Institute Inc.).

## 3 RESULTS

The characteristics of 8431 participants at visit 4 are shown in Table 1, grouped by hs‐cTnT category change between visit 2 and visit 4. Compared to participants who remained in the same category of hs‐cTnT concentrations at both visits, those who had moved up categories at visit 4 were older, more like to be male, had diabetes and hypertension treatment, had higher blood NT‐proBNP concentration level, and had a more negative ECG p wave terminal force in V1. The characteristics of all participants at visit 2 are shown in Table S1.

**TABLE 1:** Baseline characteristics from visit 4 of study participants grouped by hs‐cTnT change, ARIC study, 1996–2017

Visit 2 (1990–1992)	Undetectable		Detectable			Elevated	
Visit 4 (1996–1998)	Undetectable	Detectable & elevated	Undetectable	Detectable	Elevated	Undetectable & detectable	Elevated
*N*	3780	2042	551	1532	307	61	158
Age, years	60.9 (5.1)	63.1 (5.6)	62.5 (5.6)	64.6 (5.6)	65 (5.9)	63.9 (5.5)	65.7 (5.1)
Sex, %women	2906 (76.9)	1100 (53.9)	350 (63.5)	571 (37.3)	71 (23.1)	22 (36.1)	36 (22.8)
Race, %African American	744 (19.7)	392 (19.2)	132 (24)	339 (22.1)	78 (25.4)	17 (27.9)	63 (39.9)
Body mass index, kg/m^2^	28.2 (5.4)	29.1 (5.7)	28.7 (5.6)	29.2 (5.6)	29.7 (5.4)	29.5 (6.3)	30.2 (5.6)
Systolic blood pressure, mmHg	124.1 (17.8)	127.2 (18.5)	127.2 (18.1)	131 (19.9)	130.9 (21.1)	127 (18.8)	133.3 (19.8)
High‐density lipoprotein, mg/L	53.6 (16.4)	50.6 (17)	51.1 (16)	47.4 (15.1)	44.4 (14.3)	49 (18.1)	46.4 (15.7)
Low‐density lipoprotein, mg/L	123.8 (33.4)	121.8 (33.1)	125.7 (32.2)	123.9 (33.2)	117.5 (32.5)	129.2 (34.8)	121.3 (31.4)
ECG p wave terminal force in V1, μV ms	−2198 (1873.6)	−2583.1 (2167)	−2453.1 (2077.6)	−2610.1 (2205.3)	−2845.1 (2688.5)	−2627.6 (2130.8)	−3141.2 (2847.7)
Smoking status							
Current smoker	720 (19.1)	238 (11.7)	75 (13.6)	127 (8.3)	27 (8.8)	7 (11.5)	19 (12)
Former smoker	1423 (37.7)	908 (44.5)	203 (36.8)	727 (47.5)	150 (48.9)	31 (50.8)	80 (50.6)
Never smoker	1637 (43.3)	896 (43.9)	273 (49.6)	678 (44.3)	130 (42.4)	23 (37.7)	59 (37.3)
Drinking status							
Current drinker	2038 (53.9)	1028 (50.3)	254 (46.1)	761 (49.7)	143 (46.6)	27 (44.3)	57 (36.1)
Former drinker	979 (25.9)	609 (29.8)	158 (28.7)	426 (27.8)	105 (34.2)	21 (34.4)	73 (46.2)
Never drinker	763 (20.2)	405 (19.8)	139 (25.2)	345 (22.5)	59 (19.2)	13 (21.3)	28 (17.7)
Hypertension treatment, %	1228 (32.5)	804 (39.4)	225 (40.8)	674 (44)	171 (55.7)	27 (44.3)	104 (65.8)
Aspirin use, %	1791 (47.4)	1108 (54.3)	307 (55.7)	808 (52.7)	170 (55.4)	33 (54.1)	85 (53.8)
Statin use, %	317 (8.4)	180 (8.8)	54 (9.8)	127 (8.3)	33 (10.8)	4 (6.6)	16 (10.1)
Diabetes, %	361 (9.6)	290 (14.2)	64 (11.6)	280 (18.3)	95 (30.9)	16 (26.2)	65 (41.1)
Left Ventricular Hypertrophy, %	48 (1.3)	42 (2.1)	15 (2.7)	44 (2.9)	14 (4.6)	2 (3.3)	6 (3.8)
NT‐proBNP, pg/ml	59.85 (30.85, 108.35)	65.45 (31.2, 124.2)	59.3 (29.1, 110.9)	67 (32.5, 127.15)	87 (40.1, 178.9)	59.4 (25.1, 113.7)	74.05 (37.4, 153.9)
Triglycerides, mg/dl	121 (88, 169)	119 (87, 167)	121 (88, 164)	119 (85, 169)	120 (88, 181)	125 (92, 165)	118.5 (91, 178)
C‐reactive protein, mg/L	2.56 (1.13, 5.5)	2.23 (1.04, 5.01)	2.34 (1.1, 4.9)	1.93 (0.93, 4.92)	2.38 (1.02, 5.75)	1.92 (0.95, 4.6)	2.44 (1.12, 5.8)

*Note*: Data are shown as frequency (percentage) or mean (SD) for continuous variables of the sample; for NT‐proBNP, triglycerides and c‐reactive protein, data are shown as median(Q1, Q3).

### 3.1 Association of 6‐year change in hs‐cTnT with AF

The association between 6‐year change in hs‐cTnT and incident AF after visit 4 is shown in Table 2. During a mean follow‐up of 16.5 years after visit 4, there were 1629 incident AF events. The overall AF incidence rate was 12 per 1000 person‐years. Compared to the groups with unchanged category of hs‐cTnT concentration at both visits, the AF incidence rates in the groups with increased hs‐cTnT concentrations at visit 4 were higher, regardless of the category of hs‐cTnT at visit 2. In a fully adjusted model, the risk of AF was 1.28 times higher (95% CI 1.12–1.48) in the group that went from undetectable hs‐cTnT to detectable or elevated levels in a 6‐year time span, compared to those who remained in the undetectable group. Using the group with detectable hs‐cTnT concentration at both visits as the reference group, the group that increased from detectable at visit 2 to elevated hs‐cTnT at visit 4 had an higher risk of incident AF (HR = 1.30, 95% CI 1.01–1.68), and that in the group that decreased from detectable at visit 2 to undetectable hs‐cTnT at visit 4 had a lower risk of AF (HR = 0.74, 95% CI 0.59–0.94) in the fully‐adjusted model. Finally, those with elevated hs‐cTnT levels at visit 2 had a similar risk of incident AF, no matter what category they were in at visit 4. (HR = 1.00, 95% CI 0.51–2.00). A 1‐unit increase in the difference of log‐transformed hs‐cTnT concentrations was associated with a 43% increase in AF hazard (HR = 1.43, 95% CI 1.29–1.59).

**TABLE 2:** Association of hs‐cTnT categories with incident atrial fibrillation, ARIC study, 1996–2017

Visit 2 (1990–1992)	Undetectable		Detectable			Elevated		Increased <50%	Increased ≥50%	Continuous (ln‐transformed)
Visit 4 (1996–1998)	Undetectable	Detectable & elevated	Undetectable	Detectable	Elevated	Undetectable & detectable	Elevated			
AF cases, *N*	541	411	103	421	89	19	45	594	1035	1629
AF incidence, /1000 PYs	8	13	11	18	24	23	23	14	11	12
HR (95% CI)										
Adjusted for age, sex, race	Ref	1.39 (1.22, 1.60)	0.71 (0.56, 0.88)	Ref	1.54 (1.22, 1.93)	0.99 (0.57, 1.72)	Ref	Ref	0.94 (0.85, 1.05)	1.02 (0.94, 1.10)
Fully‐adjusted model	Ref	1.28 (1.12, 1.48)	0.74 (0.59, 0.94)	Ref	1.30 (1.01, 1.68)	1.00 (0.51, 2.00)	Ref	Ref	1.43 (1.24, 1.65)	1.43 (1.29, 1.59)

*Note*: (1) Data are shown as HR (95% CI). (2) Fully‐adjusted model adjusted for age, sex, race, body mass index (BMI), smoking status, drinking status, systolic blood pressure (SBP), low‐density lipoprotein cholesterol (LDLc), high‐density lipoprotein cholesterol (HDLc), ECG p wave terminal force in V1, triglycerides, diabetes history, ECG‐based left ventricular hypertrophy (LVH), use of anti‐hypertension medication, use of lipid lowering medications, c‐reactive protein, NT‐proBNP, eGFR, study center, and ln(visit 2 hs‐cTnT) level.

Among participants with ≥50% increase in hs‐cTnT level, the AF incidence rate was 11 per 1000 person‐years, while that among participants with less than 50% increase in hs‐cTnT level was 14 per 1000 person‐years. Hs‐cTnT concentration change was associated with AF incidence risk (Table 2). The HR among participants with ≥50% hs‐cTnT increase was 1.43 (95% CI 1.24–1.65) compared to participants with less than 50% hs‐cTnT increase, adjusting for all the covariates.

### 3.2 Improvement in AF risk prediction with hs‐cTnT change

The C‐statistics of models without and with change in hs‐cTnT and net reclassification improvement are presented in Table 3. Adding log‐transformed absolute hs‐cTnT change to an age, sex and race‐adjusted model increased the c‐statistic by 0.01, but did not increase it meaningfully in the model including the CHARGE‐AF variables (increase in c‐statistic <0.001). The user‐defined NRIs results from adding hs‐cTnT change to the established model were 0.0512 (95% CI 0.0207, 0.0586) and 0.0046 (95% CI −0.0182, 0.0308) in the two models, respectively. The continuous NRI by adding hs‐cTnT change were 0.2927 (95% CI 0.1999, 0.3837) and 0.2537 (95% CI 0.1666, 0.3396) in the two models, indicating 6‐year hs‐cTnT change improve the predictive performance with a small effect size.^29^

**TABLE 3:** Additional predictive ability of hs‐cTnT change added to risk factors for AF, ARIC study, 1996–2017

Models	C‐statistics			NRI			
	Base model	Base model + ln(hs‐cTnT change)	∆C	NRI (0.05, 0.10)	95% CI	Continuous NRI	95% CI
Adjusted for age, sex, race, hs‐cTnT level at visit 2	0.67 (0.65, 0.68)	0.68 (0.66, 0.69)	0.01	0.0512	0.0207, 0.0856	0.2927	0.1999, 0.3837
CHARGE‐AF score	0.71 (0.69, 0.72)	0.71 (0.70, 0.72)	<0.001	0.0046	−0.0182, 0.0308	0.2537	0.1666, 0.3396

*Note*: (1) CHARGE‐AF score model adjusted for age, race, height, systolic blood pressure (SBP), diastolic blood pressure (DBP), diabetes history, ECG‐based left ventricular hypertrophy (LVH), smoking status, use of anti‐hypertension medication, PR interval, hs‐cTnT level at visit 2. (2) NRI, net reclassification index.

### 3.3 Sensitivity analysis

After performing multiple imputation of the missing data, results were consistent with those of our primary analyses (Table S2). The risk of incident AF among patients with increased ≥50% hs‐cTnT was 1.36 (95% CI 1.19–1.56), and the HR for continuous log‐transformed hs‐cTnT change was 1.33 (95% CI 1.21–1.46).

## 4 DISCUSSION

Our study found that 6‐year change of hs‐cTnT concentrations was related to the risk of incident AF among ARIC cohort participants followed for 16 years. Greater hs‐cTnT change was associated with higher risk of incident AF, independent of other risk factors, including visit 2 hs‐cTnT. Additionally, individuals with an increase of hs‐cTnT over 50% had higher risk of incident AF, compared to individuals with an increase of hs‐cTnT less than 50%. However, individuals who had elevated hs‐cTnT at visit 2 had a similar risk of incident AF no matter what their hs‐cTnT levels were 6 years later. The C‐statistics and NRI did not suggest significant increase in predictive ability by adding the change of hs‐cTnT into an established model for AF prediction.

Previous studies have consistently reported associations between hs‐cTnT concentrations and risk of AF incidence. Among patients who underwent programmed cardiac surgery with cardiopulmonary bypass, high presurgical hs‐cTnT levels were independently predictive of patients developing AF after cardiac surgery.^30^ Previously in the ARIC study, we reported the risk of incident AF increased by 16% with a one standard deviation increase of ln(hs‐cTnT) level.^15^ In the Cardiovascular Health Study, a large prospective cohort of ambulatory older adults, hs‐cTnT concentrations were significantly associated with incident AF beyond traditional risk factors.^16^ Notably, in these two large, general population‐based cohort studies, other established risk factors such as hypertension and smoking status were adjusted for in the model, suggesting hs‐cTnT levels were associated with risk of AF incidence independent of established risk factors.^31^ However, inconsistent results have also been observed. Among 883 individuals in the Uppsala Longitudinal Study of Adult Men (ULSAM) and 978 individuals in the Prospective Investigation of the Vasculature in Uppsala Seniors (PIVUS) study, the association between hs‐cTnT and incident AF was attenuated after controlling for NT‐proBNP in the model.^32^

To our knowledge, however, little is known regarding the association between long‐term circulating hs‐cTnT change with incident AF risk, as well as underlying pathophysiological mechanisms. The ARIC study has previously reported that 6‐year change in hs‐cTnT was independently associated with death, HF, and incident CHD.^22^ Recently, in the Nord‐Trøndelag Health (HUNT) Study, the largest population‐based cohort in Norway, investigators examined the temporal changes in cardiac troponin I (hs‐cTnI) with risk of cardiovascular events in the general population.^33^ They found that both relative and absolute increases in hs‐cTnI levels were independently associated with cardiovascular risk. Hs‐cTnT and hs‐cTnI are moderately correlated and share some characteristics in predicting AF,^34^ so these findings suggested that temporal change in hs‐cTnT could be also associated with cardiovascular risk. Since AF and other cardiovascular diseases are related to each other,^35^ long‐term change in hs‐cTnT might provide predictive information on the risk of overall cardiovascular disease among patients with AF.^36^

Despite the observed association between changes in hs‐cTnT concentration and AF risk, this information did not increase the predictive ability of the CHARGE‐AF score, a well‐established model for the prediction of AF. This observation is in contrast with our previous analysis of the ARIC cohort focusing in NT‐proBNP changes, which described improvements in the C‐statistic for AF prediction after adding information on repeated NT‐proBNP measurements.^21^

Our study has several strengths. First, the ARIC study recruited a large and diverse population. Second, we took advantage of the repeated measurements of hs‐cTnT in samples obtained several years apart. Third, in the ARIC study, we were able to adjust for other biomarkers, particularly NT‐proBNP. Our study also has some limitations. First, the study sample were limited to whites and African Americans who were recruited from four communities in the Unites States, which might not be generalizable to other populations. Second, we were not able to differentiate between AF patterns, such as paroxysmal AF, persistent AF, or permanent AF. Third, the method of AF ascertainment may have resulted in missing asymptomatic cases as well as those exclusively managed in outpatient settings. However, we have demonstrated adequate validity of our approach to case ascertainment and incidence rates of AF in the ARIC cohort are similar to those reported in other community‐based studies with more intensive methods for AF identification.^26^ Finally, as an observational study, there might be residual and unmeasured confounding that potentially could explain the association.

In conclusion, we have found that a 6‐year change in mid‐life hs‐cTnT concentrations were associated with risk of incident AF in a community‐based population. The association remains significant after adjusting for other established risk factors. Change in hs‐cTnT, however, did not contribute meaningfully to the prediction of AF. These results highlight the role that progression in myocardial injury could have in the development of the AF substrate, but do not support the use of repeated hs‐cTnT measures for prediction of AF. Further investigations regarding the association in other population are needed to verify and identify high‐risk individuals.

## CONFLICT OF INTEREST

The authors declare no conflict of interest.

## Supporting information


**TABLE S1**: Visit 2 characteristics of study participants grouped by hs‐cTnT change, ARIC study, 1996–2017.
**TABLE S2**: AF incidence after visit 4 according to the categories of hs‐cTnT change between visit 2 and visit 4. Model using multiple imputation by chained equation (MICE), adjust for losses to follow‐up between visit 2 and visit 4. ARIC Study, 1996–2017.

## Data Availability

The authors confirm that all data underlying the findings are fully available without restriction. The repository can be accessed online (https://biolincc.nhlbi.nih.gov/studies/aric/?q=aric).
