# Supplementary material for: 6‐year change in high sensitivity cardiac troponin T and the risk of atrial fibrillation in the Atherosclerosis Risk in Communities cohort
Source: Clin Cardiol. 2021 Sep 21;44(11):1594–601. doi: 10.1002/clc.23727 (PMC8571551; doi:10.1002/clc.23727)
Supplement: Supplementary file 1 — TABLE S1: Visit 2 characteristics of study participants grouped by hs‐cTnT change, ARIC study, 1996–2017. TABLE S2: AF incidence after visit 4 according to the categories of hs‐cTnT change between visit 2 and visit 4. Model using multiple imputation by chained equation (MICE), adjust for losses to follow‐up between visit 2 and visit 4. ARIC Study, 1996–2017. [file CLC-44-1594-s001.docx]

| **Supplemental Table 1. Visit 2 characteristics of study participants grouped by hs-cTnT change, ARIC study, 1996-2017** | | | | | | | |
| --- | --- | --- | --- | --- | --- | --- | --- |
| Visit 2 (1990-92) | undetectable | | detectable | | | elevated | |
| Visit 4 (1996-98) | undetectable | detectable&elevated | undetectable | detectable | elevated | undetectable&detectable | elevated |
| N | 3780 | 2042 | 551 | 1532 | 307 | 61 | 158 |
| Age,years | 54.8(5.1) | 57.1(5.6) | 56.5(5.6) | 58.6(5.6) | 58.9(5.8) | 57.9(5.5) | 59.7(5.1) |
| Sex, %women | 2906(76.9) | 1100(53.9) | 350(63.5) | 571(37.3) | 71(23.1) | 22(36.1) | 36(22.8) |
| Race, %African American | 744(1976) | 392(19.2) | 132(24.0) | 339(22.1) | 78(25.4) | 17(27.9) | 63(39.9) |
| Body mass index, kg/m^2^ | 27.1(5.1) | 28.1(5.2) | 27.7(5.3) | 28.4(5.3) | 29(5.2) | 29.1(6.3) | 29.7(5.5) |
| Systolic blood pressure,mmHg | 116.2(16.3) | 120.3(16.8) | 119.3(16.5) | 123.4(17.6) | 126.8(20.1) | 122.6(21) | 130.3(21.1) |
| High density lipoprotein, mg/L | 54.5(16.8) | 49.5(16.6) | 52.4(17) | 47.4(15.2) | 43.7(13.4) | 45.6(15.6) | 45.2(14.4) |
| Low density lipoprotein, mg/L | 130.9(35.8) | 131.7(35.2) | 134.5(35) | 134.1(36.2) | 130.9(37.2) | 137.7(57.4) | 133.9(37.7) |
| ECG p wave terminal force in V1, µV*ms | -1682.5(1657.4) | -2001.4(1874) | -1923(1807.6) | -2156.2(1945.5) | -2304.3(2164) | -2501.3(2156.2) | -2670.3(2518) |
| Smoking Status |  |  |  |  |  |  |  |
| Current smoker | 870(23.0) | 350(17.1) | 86(15.6) | 195(12.7) | 40(13.0) | 9(14.8) | 30(19.0) |
| Former smoker | 1242(32.9) | 803(39.3) | 197(35.8) | 654(42.7) | 138(45.0) | 31(50.8) | 68(43.0) |
| Never smoker | 1668(44.1) | 889(43.5) | 268(48.6) | 683(44.6) | 129(42.0) | 21(34.4) | 60(38.0) |
| Drinking Status |  |  |  |  |  |  |  |
| Current drinker | 2308(61.1) | 1219(59.7) | 307(55.7) | 905(59.1) | 173(56.4) | 33(54.1) | 68(43.0) |
| Former drinker | 624(16.5) | 379(18.6) | 114(20.7) | 262(17.1) | 70(22.8) | 14(23.0) | 62(39.2) |
| Never drinker | 848(22.4) | 444(21.7) | 130(23.6) | 365(23.8) | 64(20.9) | 14(23.0) | 28(17.7) |
| Hypertension treatment, % | 809(21.4) | 549(26.9) | 158(28.7) | 477(31.1) | 129(42.0) | 25(41.0) | 77(48.7) |
| Diabetes, % | 259(6.9) | 226(11.1) | 53(9.6) | 228(14.9) | 70(22.8) | 12(19.7) | 49(31.0) |
| Left Ventricular Hypertrophy, % | 33(0.9) | 30(1.5) | 10(1.8) | 42(2.7) | 6(2.0) | 1(1.6) | 12(7.6) |
| NT-proBNP, pg/mL | 51.02(27.53, 86) | 46.7(26, 82.79) | 48.97(24.57, 88.63) | 44.75(23.85, 81.06) | 50.94(25.91, 97.81) | 50.73(22.68, 116.8) | 47.54(26.06, 100.4) |
| Triglycerides, mg/dL | 106(78, 147) | 112(81, 157) | 111(81, 154) | 115(80.5, 163) | 116(82, 183) | 124(97, 140) | 115(80, 165) |
| c-reactive protein, mg/L | 2.03(0.95, 4.34) | 2.01(0.96, 4.2) | 1.98(1, 4.24) | 1.91(0.94, 4.02) | 1.8(0.99, 3.86) | 2.17(0.95, 5.52) | 2.36(1.19, 4.93) |
| Notes: Data are shown as frequency (percentage) or mean (SD) for continuous variables of the sample; for NT-proBNP, triglycerides and c-reactive protein, data are shown as median(Q1, Q3). | | | | | | | |

| **Supplemental Table 2. AF incidence after visit 4 according to the categories of hs-cTnT change between visit 2 and visit 4. Model using multiple imputation by chained equation (MICE), adjust for losses to follow-up between visit 2 and visit 4. ARIC Study, 1996-2017** | | | | | | | | | | |
| --- | --- | --- | --- | --- | --- | --- | --- | --- | --- | --- |
| Visit 2 | undetectable | | detectable | | | elevated | | increased<50% | Increased≥50% | continuous |
| Visit 4 | undetectable | detectable&elevated | undetectable | detectable | elevated | undetectable&detectable | elevated |  |  |  |
| AF incidence | 8 | 13 | 11 | 18 | 22 | 21 | 23 | 14 | 11 | 12 |
| HR |  |  |  |  |  |  |  |  |  |  |
| Adjusted for age, sex, race | ref | 1.40  (1.23, 1.59) | 0.71  (0.58, 0.87) | ref | 1.45  (1.17, 1.81) | 0.89  (0.52, 1.52) | ref | ref | 0.95  (0.86, 1.05) | 1.02  (0.95, 1.10) |
| Fully-adjusted model | ref | 1.31  (1.14, 1.49) | 0.73  (0.59, 0.91) | ref | 1.23  (0.97, 1.57) | 0.99  (0.51, 1.92) | ref | ref | 1.36  (1.19, 1.56) | 1.33  (1.21, 1.46) |
| Notes: 1) Data are shown as HR(95% CI) 2)Fully-adjusted model adjusted for age, sex, race, body mass index (BMI), smoking status, drinking status, systolic blood pressure (SBP), low-density lipoprotein cholesterol (LDLc), high-density lipoprotein cholesterol (HDLc), ECG p wave terminal force in V1, triglycerides, diabetes history, ECG-based left ventricular hypertrophy (LVH), use of anti-hypertension medication, use of lipid lowering medications, c-reactive protein, NT-proBNP, eGFR, study center and ln(visit 2 hs-cTnT) level. 3) Multiple imputation by chained equation was used. | | | | | | | | | | |
